# Preparation of Edible Non-wettable Coating with Soybean Wax for Repelling Liquid Foods with Little Residue

**DOI:** 10.3390/ma13153308

**Published:** 2020-07-24

**Authors:** Tianyu Shen, Shumin Fan, Yuanchao Li, Guangri Xu, Wenxiu Fan

**Affiliations:** School of Chemistry and Chemical Engineering, Henan Institute of Science and Technology, Xinxiang, Henan 453003, China; xxttyx@163.com (T.S.); liyuanchaozzu@126.com (Y.L.); xugr70@163.com (G.X.)

**Keywords:** non-wettable coating, edible, soybean wax, residue

## Abstract

Liquid food adhesion on containers has increased food waste and pollution, which could be effectively alleviated with a superhydrophobic surface. In this research, the superhydrophobic coating was fabricated with edible soybean wax on different substrates by a spraying method. The coated surface showed excellent superhydrophobicity due to its microstructure formed by self-roughening, which could repel a variety of viscous liquid food with the apparent contact angle of 159 ± 2°. The coated surface was still liquid-repellent after hot water immersion (45 °C), abrasion test with sandpaper, water impact, finger touch and immersion into yogurt. The liquid-repellent coating with soybean wax, which is natural and green, is promising for application in the food industry to reduce waste.

## 1. Introduction

With the development of the economy, people’s living standards improved, especially in food demand. In daily life, viscous liquid food (such as yogurt, honey, milk, coffee, et al.) residue remain adhering to the container after drinking, which has given us a great deal of inconvenience and resulted in huge waste (up to 15% of liquid food products) [1]. Food adhesion problems could be effectively alleviated by using a superhydrophobic surface. Superhydrophobic surfaces have become a popular topic due to their application in oil/water separation [2,3,4], anti-corrosion [5], drag reduction [6], reproductive medicine and cryobiology [7] et al. Superhydrophobic surfaces, characterized by high apparent contact angles (> 150°) and low contact angle hysteresis (the difference between the advancing and receding contact angles, which could lead to a low sliding angle), have tremendous practical applications, including self-cleaning and drag reduction. The non-wetting coatings could eliminate liquid food residue, avoiding the adhesion of viscous liquid to packaging material [8,9,10,11,12,13,14].

Superhydrophobic coatings are fabricated through surface texture and low solid surface energy [15] or modification of surface microstructure without using low surface-energy reagents [16,17]. Microscale structure, nanoscale structure and hierarchical structure turned out to be important for surface texture fabrication. So far, most of the studies to fabricate water-repellent surfaces use nanoparticles combined with fluorine-containing reagents. Vahabi et al. [18] prepared flexible non-wettable films by creating coatings of polyurethane and fluorinated silica particles on a substrate, which repelled varieties of liquids. Pan et al. [19] fabricated superhydrophobic stainless-steel wire meshes by creating coatings of cross-linked poly(dimethylsiloxane) and fluorodecyl polyhedral oligomeric silsequioxane. The fluorocarbon materials could decompose to perfluorooctanoic acid (PFOA), whose toxicity is biocumulative and persistent to humans. Thus, the fluorocarbon materials, classified as contaminants, are unfit for the preparation of edible non-wettable coatings.

To make a non-wettable coating on packaging material, the most important factor is safety: not allowing toxicity when contacting food. A superhydrophobic surface could be prepared by spraying a mixture of a non-polar compound and solvent [20,21]. Wang et al. [22] prepared water-repellent coatings using beeswax and carnauba wax. The coatings were non-wettable towards different liquid food, which was edible and natural. Liu et al. [23] created liquid-repellent coatings using rice bran wax and candelilla wax by a one step spraying method on polypropylene (PP) substrates, which could be used for eliminating liquid waste of food containers. Zhang et al. [24] fabricated a superhydrophobic paper surface with good transparency and stability properties via mixture coating of beeswax and carnauba wax. The wax mixture was emulsified and coated on a paper surface, followed by annealing at different temperatures. Submicrometer structure was generated on the base of micrometer spherical wax particles. Yang et al. [25] prepared three kinds of edible superhydrophobic surfaces by fumigating lard, food grade paraffin and beeswax on calcined Fe foil. All samples showed excellent superhydrophobicity and effectively repelled starch slurries and liquid foods without observable residues. The used sample could be recovered by re-fumigation. These researches have promoted the preparation of edible non-wettable materials, which should be environmentally friendly and should not contain toxic chemicals and unapproved additives. 

Soybean wax is made from natural soybeans, which are abundant. Thus, the soybean wax is cheap, natural and biodegradable without environmental pollution, which has great advantages in terms of health and environmental protection [26,27]. The main ingredient of soybean wax is triacylglyceride composed of stearic acid. Herein, superhydrophobic coatings were fabricated with soybean wax by spray coating of a wax suspension in ethanol. This low-cost and simple non-wettable coating exhibited excellent liquid repelling properties against a variety of non-Newtonian viscous food liquids or hot water solution on different food packaging materials (glass, paper, plastic and ceramics). The coating provided excellent resistance to hot water immersion, sandpaper abrasion, water impact, finger touching and yogurt immersion. Thus, the non-wettable coating with soybean wax could be used to reduce liquid food residue on different substrates, including glass slide, paper, plastic and ceramic.

## 2. Materials & Methods

### 2.1. Materials 

Ethanol was purchased from Tianjin Hengxing Chemical Reagent Manufacturing co, LTD, Tianjin, China. Soybean wax was purchased from Hubei Xinghe Chemical co, LTD, Hubei, China. Milk, fruit juice, Coca Cola, honey and black tea were purchased from local supermarkets, Xinxiang, China. Glass slides, paper, plastic and ceramic were available at local markets, Xinxiang, China.

### 2.2. Preparation of Soybean Wax Coating

**Figure 1:**
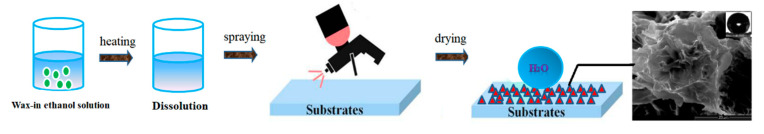
Illustration for preparation of a non-wettable surface.

As shown in Figure 1, the soybean wax suspension was prepared thorough mixture of 1 g of soybean wax and 50 mL ethanol solution followed by heating at 65 ℃ for 3 min. The hot wax suspension was sprayed (Flying Boat Glass Co. LTD, Yancheng, China) onto glass slides (Zhejiang Pride Electric Appliance Co. LTD, Jinhua, China) with a distance of 50 cm. Finally, the coated surface was dried at room temperature to obtain its superhydrophobic characteristics.

### 2.3. Characterization

The surface structure of the superhydrophobic coating was observed by a Quanta 200 scanning electron microscope (FEI, Hillsboro, OR, USA) operated at an acceleration voltage of 20 kV. The roughness of the superhydrophobic coating was analyzed by GTK-16-0300 white light interferometer (BRKR, Billerica, Massachusetts, USA). The wettability of the superhydrophobic surface was tested with an optical contact angle measuring instrument (Shenzhen testing equipment CO., LTD, Shenzhen, China, TST-200H). Apparent contact angles of the superhydrophobic coating were measured with a deionized water droplet (Shenzhen testing equipment CO., LTD, Shenzhen, China) of 10 μL on a video optical contact angle system to measure the resistance of superhydrophobic coating to different liquid foods at room temperature. The values of the apparent contact angle and the sliding angle were determined by averaging values measured at 5 different points on each sample surface.

## 3. Results

### 3.1. Morphology of the Soybean Wax Coating

**Figure 2:**
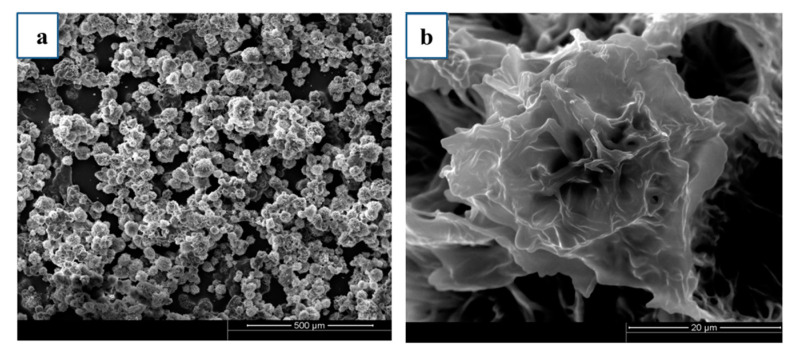
(**a**) SEM image of the soybean wax coating and (**b**) its higher magnification image.

Glass is very common in our life and it is widely used as a liquid food packaging material. After treated with soybean wax in ethanol, the surface was made up of irregular and discernible nanoscale sheets of waxy crystals, which distributed neatly and tightly (Figure 2a). At higher magnifications, a flower-like micro-nano roughness structure (Figure 2b) could be observed. The rapid volatilization of the solvent resulted in the rough structure of the coating.

**Figure 3:**
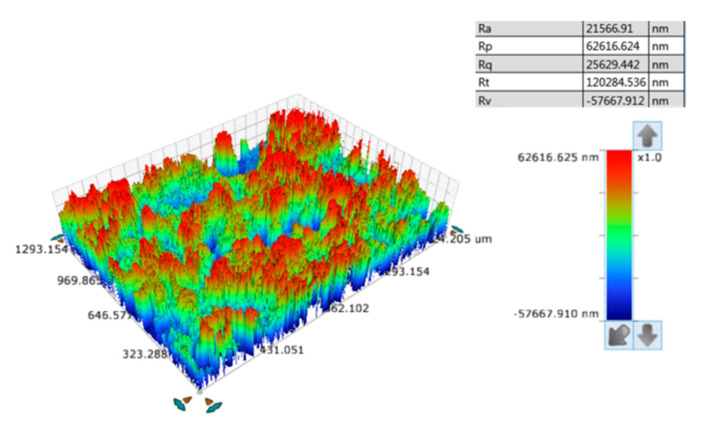
The roughness of the coating.

Futhermore, the roughness of the coating was analyzed by white light interferometer at room temperature. As shown in Figure 3, the convex hulls showed micron fluctuation structure. The staggered convex structure and uniform distribution of convex hulls on the surface facilitated its superhydrophobic characteristics.

### 3.2. Influence of Surface Density

**Figure 4:**
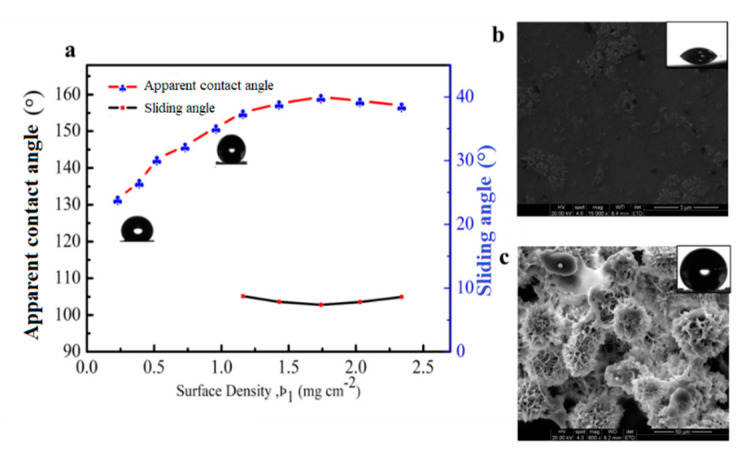
(**a**) The effect of surface density (ρ) on apparent contact angle and sliding angle; (**b**) SEM image of pristine glass and (**c**) the coated glass with ρ of 1.74 mg cm^−2^.

Figure 4a shows the influence of surface density (ρ) of soybean wax on the values of apparent contact angle and sliding angle. The water droplets stick to the coating surface when ρ is smaller than 0.87 mg cm^−2^. The maximum apparent contact angle of 159° and the minimum sliding angle of 7° were obtained with ρ of 1.74 mg cm^−2^. At ρ = 1.74 mg cm^−2^, the advancing and receding contact angle were measured as 159° and 154°, respectively, which led to low contact angle hysteresis. The high apparent contact angle and low contact angle hysteresis were from complete coverage of the coating and the desired roughness. The increase in surface density increased the thickness of the soybean wax coating, which could not change the roughness significantly. Thus, the value of apparent contact angle and sliding angle basically tended to be stable when ρ continued to increase. As shown in Figure 4b, the pristine glass substrates show a smooth surface. However, the hierarchical microscale and nanoscale roughness structure on glass surface after soybean wax coating treatment was formed (Figure 4c), which is the critical points for non-wetting [28]. Consequently, the superhydrophobic coating with soybean wax was fabricated with sufficiently high surface density of the wax.

### 3.3. Repellency to Viscous Liquid Food in Daily Life

**Figure 5:**
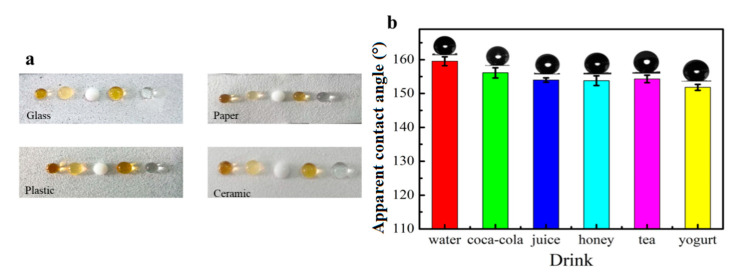
(**a**) Photographs of Coca Cola, black tea, yogurt, honey and water droplets on different coated materials and (**b**) the apparent contact angles and sliding angles of different liquid food on coated glass.

The soybean wax coating method could be easily applied on different food packaging materials, such as glass, paper, plastic and ceramic substrates. Figure 5a shows the photographs of Coca Cola, black tea, yogurt, honey and water on different soybean wax treated materials. All these materials became non-wetting with spherical shape of various liquid food droplets on the coated substrates. Figure 5b shows the as-coated glass surface had apparent contact angles of 159°, 157°, 154°, 153°, 156° and 152°, respectively to water, Coca Cola, juice, honey, tea and yogurt. The complex flow characteristics of non-Newtonian fluids like honey and yogurt resulted in a little bit lower apparent contact angle than other food liquids. The non-Newtonian fluids could have a certain “memory effect” and make it difficult to flow. As shown in Video S1, the water droplet falls down on the coating surface of glass and it bounced back to the air. When the water column quickly hit the coating surface, water droplets bounced high and rolled down along the surface (Video S2).

### 3.4. Heat Resistance

**Figure 6:**
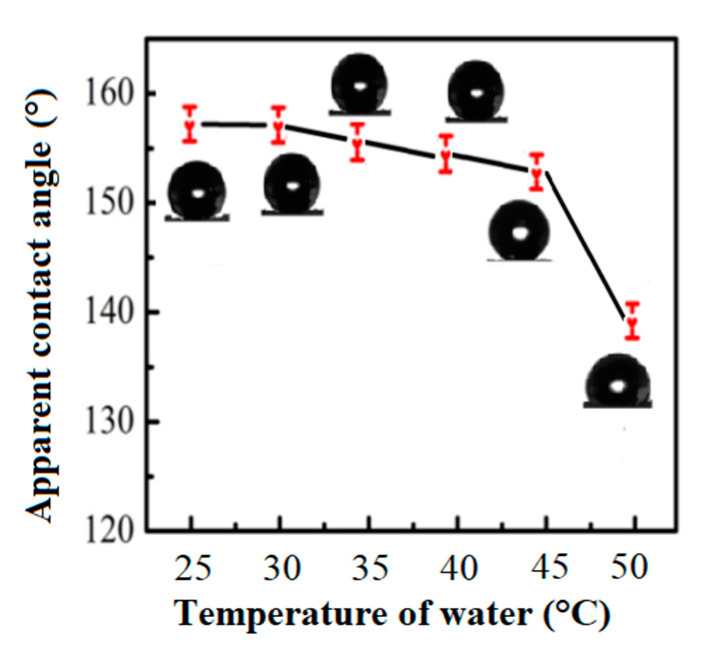
The apparent contact angles with different temperatures of immersion water.

The heat resistance of the coated surface was studied by immersing the treated glass in water heated by electric-heated thermostatic water bath (Spring Instrument. Co., LTD, Jintan, China) for 10 min. After contacting with hot water from 25 to 50 °C, the apparent contact angle was determined every 5 °C. As shown in Figure 6, The coated surface kept superhydrophobic when the water bath increased from 25 °C to 45 °C. When the water temperature increased to 50 °C, the coated surface lost its superhydrophobicity with apparent contact angle lower than 150°. The soybean wax, with the melting point of 52 °C, is a kind of phase change material. The partial disappearance of the rough structure happened when the temperature was close to 50 °C, which reduced the apparent contact angle of droplet on the surface. More research is needed to improve the robustness of soybean wax towards hot water above 50°C, which is an important factor for its practical applications.

### 3.5. Robustness and Durability of the Coating

**Figure 7:**
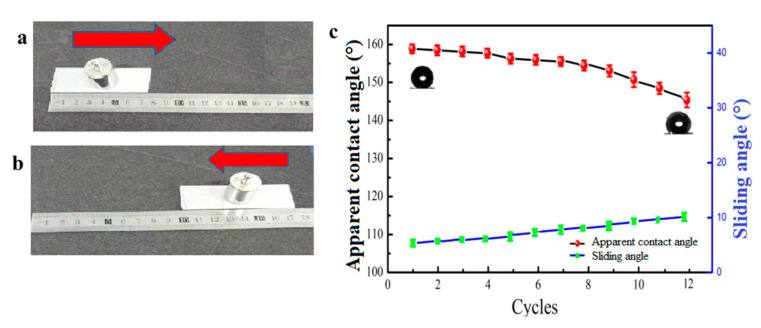
(**a**,**b**) One abrasion test cycle and(**c**) the change of apparent contact angle and sliding angle after cycles of abrasion.

**Figure 8:**
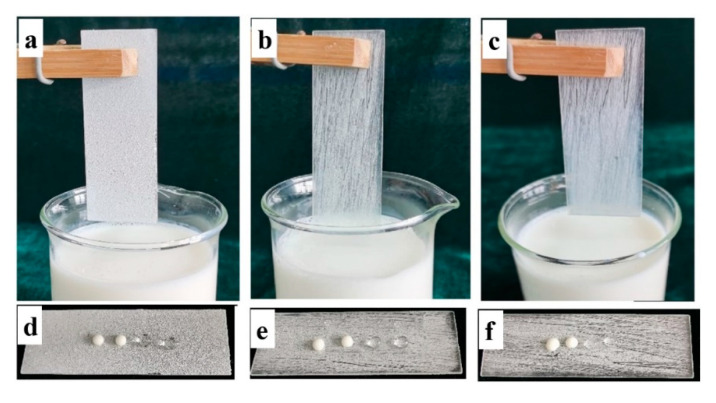
The coated glass after immersion into yogurt after (**a**) 0; (**b**) 10 and (**c**) 12 cycles of abrasion test; Photographs of yogurt and water droplets on a coated glass surface after (**d**) 0; (**e**) 10 and (**f**) 12 cycles of abrasion test.

The physical damage of viscous liquid food and external pressure could wear down the food packaging material. The physical damages of non-wettable coating contacting with viscous liquid is a big challenge for large-scale application. Robustness still remains a major challenge for application of superhydrophobic coating. Thus, the abrasion resistance of the coating was analyzed by placing the coated glass face down to a sandpaper with 1000 meshes under a weight of 100 g along a ruler for a distance of 20 cm (Figure 7a,b), which was defined as one test cycle. The change of apparent contact angle and sliding angle after cycles of abrasion is shown in Figure 7c. The coating was still superhydrophobic even after 10 cycles of abrasion. Then, the apparent contact angle decreased to less than 150°. The photographs of yogurt and water droplets on the coated glass surface after 0, 10 and 12 cycles of abrasion test are shown in Figure 8d–f. After 10 times of abrasion, the yogurt and water droplets could still maintain spherical shape on the surface. The coated glass was immersed into yogurt to see its non-wettable property. The yogurt flowed down easily without wetting or contaminating the surface after 10 times of abrasion (Figure 8b). However, the yogurt adhered to the surface due to loss of superhydrophobicity after 12 cycles of abrasion (Figure 8c). During the test, the surface was abraded longitudinally. The physical abrasion led to removal of soybean wax and partial loss of surface roughness, which were key factors for its superhydrophobicity. 

**Figure 9:**
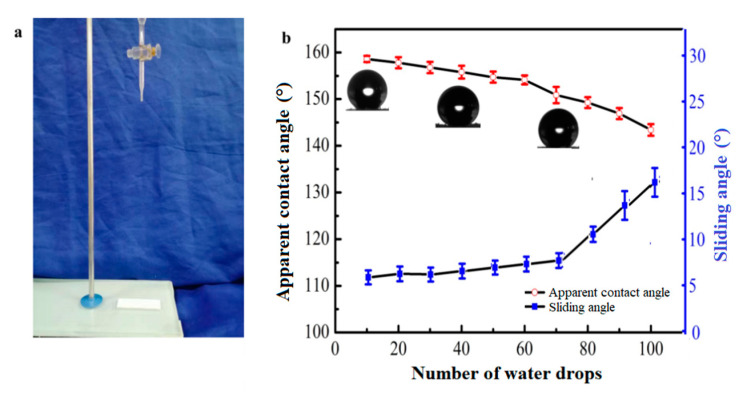
(**a**) The device used for an impact resistance test; (**b**) the change of apparent contact angle and sliding angle after water impact.

Apart from sandpaper abrasion, the impact resistance of the soybean wax coated surface was evaluated by water dropping from a height of 30 cm (Figure 9a). The apparent contact angle and sliding angle were measured every 10 drops. The change of apparent contact angle and sliding angle after water impact are shown in Figure 9b. The coating maintained excellent water repellency after 70 drops with excellent superhydrophobicity. Then, the apparent contact angle decreased to less than 150°. The impact resistance of the coated surface was crucial for its application in liquid packaging.

**Figure 10:**
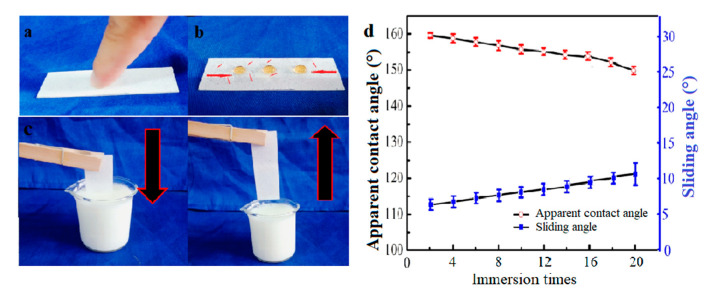
(**a**) The durability test by pressing with a finger; (**b**) photograph of water droplets after touching; (**c**) one-time immersion in yogurt and (**d**) the change of apparent contact angle and sliding angle of the treated surface plotted against immersion times in yogurt.

**Figure 11:**
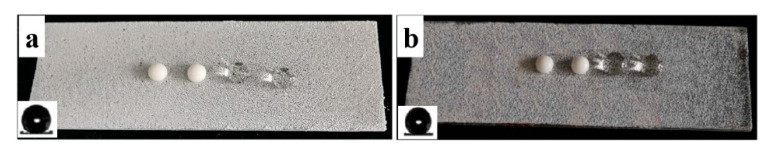
Photographs of yogurt and water droplets (**a**) on a coated glass surface and (**b**) after 200 days.

The coated surface was pressed with a finger to test its durability (Figure 10a). The direction of touching is marked with a red line in Figure 10b. The water droplets maintained spherical shape; they could roll randomly on the touched surface. As shown in Figure 10c, the coated glass was immersed into yogurt repeatedly. The superhydrophobicity of the treated surface was studied after immersion (Figure 10d). The apparent contact angle decreased and the sliding angle increased with the number of immersion times. After immersed into yogurt for 20 times, the coating remained superhydrophobic. The coated glass was immersed into yogurt for 24 h to study the effect of immersion time on the superhydrophobicity of the coating. After a 24 h immersion, the apparent contact angle of the coated surface was still above 150°, showing excellent liquid-repellent properties. To further evaluate the durability of the coated surface, the glass treated with soybean wax was exposed to air for 200 days. As shown in Figure 11b, the coating remained superhydrophobic with the apparent contact angle higher than 150°. In the meantime, a water droplet could still maintain its spherical shape on the surface, demonstrating the stability of the coating exposed to air. The soybean wax coating could maintain stability because of its long chain alkane composition exposed to a corrosive environment.

### 3.6. Comparison with Other Coatings

It is reported that the edible superhydrophobic coating was fabricated by paraffin wax, beeswax, microcrystalline wax and carnauba wax, et al. The apparent contact angle and sliding angle of the coating treated by soybean wax was compared with other waxes reported in the existing literature. Different from microcrystalline wax and paraffin wax, the soybean wax, beeswax, carnauba wax and rice bran wax are renewable waxes. As shown in Table 1, the apparent contact angles of the coating with beeswax, microcrystalline wax, mixture of candelilla wax and rice bran wax were slightly higher than that of soybean wax, used in our research. The different superhydrophobic properties of waxes are owed to their different chemical constitution and dissolution-precipitation character in solvent. The soybean wax has advantages of wide source and low price. Thus, it is promising for application in the food industry, to reduce waste.

The coating with soybean wax was subjected to abrasion tests with sandpaper, water impact, finger touch and immersion into yogurt to evaluate its robustness and durability. Zhao et al. [29] immersed the superhydrophobic coating of paraffin wax into 1M HCl (Sinopharm Chemical Reagent CO. LTD, Shanghai, China), 1M NaOH (Sinopharm Chemical Reagent CO. LTD, Shanghai, China) and deionized water made by water purification machine (Ruide Chemical Instrument CO. LTD, Zaozhuang, China), respectively. After 24 h of immersion, the apparent contact angles of the treated coatings were all higher than 150°. Liu et al. [23] created an edible liquid-repellent coating by mixing rice bran wax and candelilla wax on polypropylene substrates. The apparent contact angle of the coating lost its superhydrophobic properties after 8 cycles during the sandpaper abrasion test. After 1200 cycles of 180° bending, the coating was still non-wettable without any change in appearance. The edible coating with soybean wax in our research could endure 10 cycles of abrasion test under the same pressure. Chen et al. [30] used the solution-dipping method for sequential deposition of a trilayer of branched poly(ethylenimine), ammonium polyphosphate and fluorinated-decyl polyhedral oligomeric silsesquioxane. The superhydrophobic coating could maintain its flame-retardant and self-healing properties even after 1000 cycles of abrasion (under a pressure of 44.8 kPa). However, the non-wettable coatings made of fluorocarbon materials are not suitable for the application of direct contact with food. Thus, the edible superhydrophobic coatings need to improve their heat resistance and robustness, which is also our future research direction.

### 3.7. Application Test

**Figure 12:**
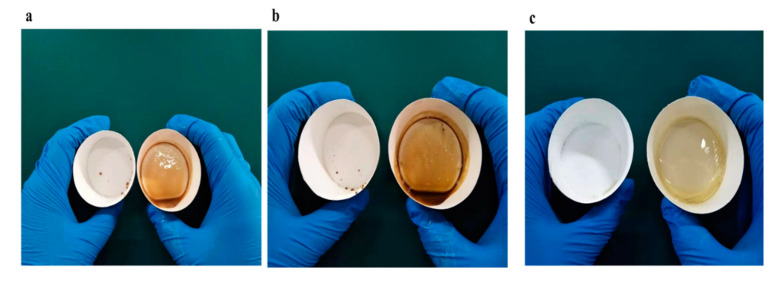
Photographs of (left) remaining viscous liquid after pouring in a soybean wax coated cup and (right) original cup: (**a**) milky tea; (**b**) chocolate syrup and (**c**) honey.

To demonstrate the practicality of the edible superhydrophobic coating of soybean wax, the inside of a paper cup was coated with soybean wax to test its performance. The cups were filled with various viscous liquid food, including milky tea, honey and chocolate syrup. As shown in Video S3, the liquid was poured out from the cup. The high-viscous liquid in the soybean wax treated cup came out easily with a little residue, demonstrating liquid-repellent properties of the paper cup. In contrast, without coating treatment, it was hard for the liquid to flow out and significant residues were left stuck to the original cup. Figure 12 shows the photographs of the remaining viscous liquid after pouring. There is hardly any liquid left with the coated cup. Thus, the edible non-wettable coating of soybean wax could repel complex non-Newtonian fluids with little residue. 

## 4. Conclusions

In this work, an edible superhydrophobic coating was prepared with soybean wax by a one-step method, which could be widely applied to various packaging materials to eliminate liquid food residues and save resources. Through a variety of tests, the coating showed excellent stability to repel high-viscosity non-Newtonian liquid food and even a hot aqueous solution (about 45 °C). The coating maintained its superhydrophobicity after abrasion tests with sandpaper, water impact, finger touch and immersion into yogurt. The superhydrophobic coating prepared by soybean wax was edible and robust; it could be widely used to reduce liquid food residues due to its simple, renewable and low-cost characteristics. 

## Figures and Tables

**Table 1:** The apparent contact angle and sliding angle of the superhydrophobic coatings with different waxes.

Waxes	Apparent Contact Angle (°)	Sliding Angle (°)
Soybean Wax (This Research)	159 ± 2	7 ± 1
Paraffin Wax [29]	158 ± 3	7 ± 1
Beeswax [29]	162 ± 2	7 ± 1
Microcrystalline Wax [29]	161 ± 2	5 ± 1
Carnauba Wax [29]	150 ± 2	22 ± 2
Mixture of Candelilla Wax and Rice Bran Wax [23]	162 ± 2	1 ± 1

## Supplementary Materials

The following are available online at https://www.mdpi.com/1996-1944/13/15/3308/s1. Video S1: The bounce of a water droplet on the coating surface of glass. Video S2: The hitting of a water column on the coating surface. Video S3: The remaining viscous liquid after pouring in (left) soybean wax coated cup and (right) original cup.
