# Supplementary material for: Preparation of Edible Non-wettable Coating with Soybean Wax for Repelling Liquid Foods with Little Residue
Source: Materials (Basel). 2020 Jul 24;13(15):3308. doi: 10.3390/ma13153308 (PMC7435775; doi:10.3390/ma13153308)
Supplement: Supplementary file 1 [file materials-13-03308-s001.zip › materials-853907-supplementary checked.pdf]

materials

MDPI

Supplementary Materials

Preparation of Edible Non-wettable Coating with Soybean Wax for Repelling Liquid Foods with Little Residue

Tianyu Shen, Shumin Fan *, Yuanchao Li, Guangri Xu and Wenxiu Fan *

School of Chemistry and Chemical Engineering, Henan Institute of Science and Technology, Xinxiang, Henan 453003, China; xxttyx@163.com (T.S.); liyuanchaozzu@126.com (Y.L.); xugr70@163.com (G.X.)

* Correspondence: fansm88@hotmail.com (S.F.); fwxiu@hist.edu.cn (W.F.)

Received: 17 June 2020; Accepted: 20 July 2020; Published: 24 July 2020

Video S1: The bounce of water droplet on the coating surface of glass.

Video S2: The hitting of water column on the coating surface.

Video S3: The remaining of viscous liquid after pouring in soybean wax coated cup (left) and original cup (right).
